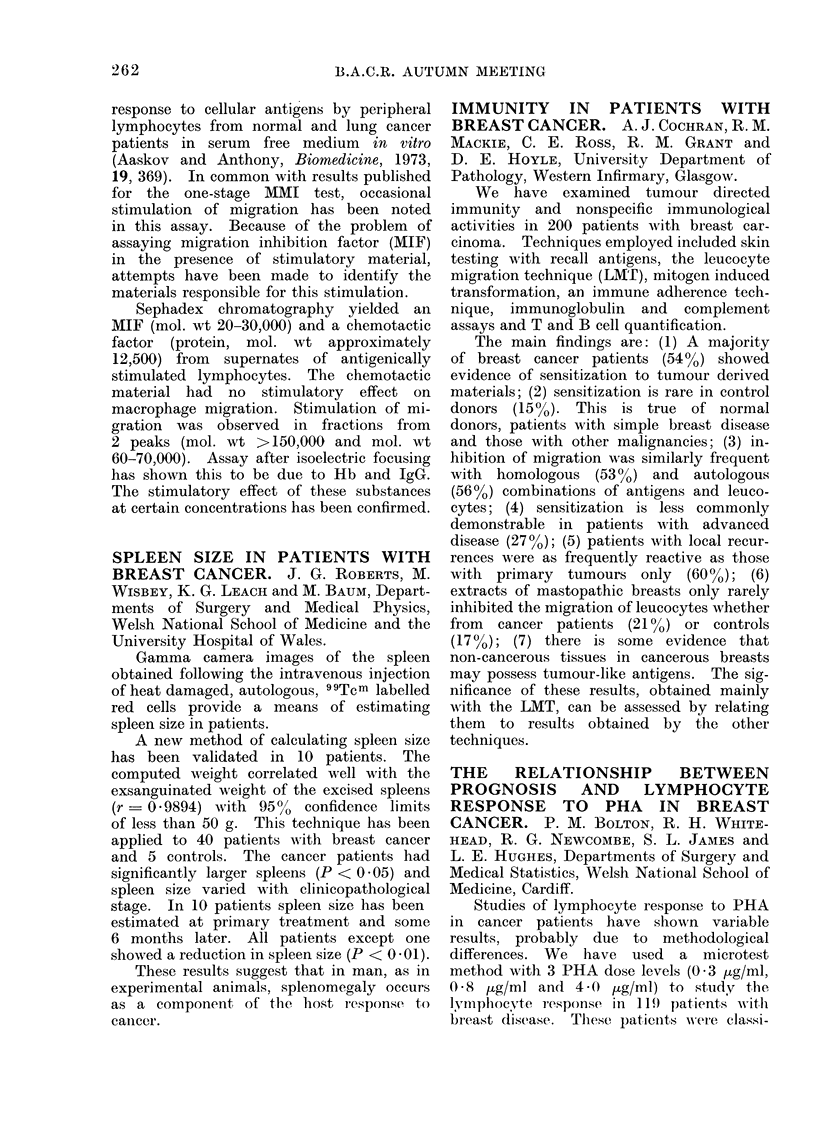# Proceedings: Immunity in patients with breast cancer.

**DOI:** 10.1038/bjc.1975.43

**Published:** 1975-02

**Authors:** A. J. Cochran, R. M. Mackie, C. E. Ross, R. M. Grant, D. E. Hoyle


**SPLEEN SIZE IN PATIENTS WITH BREAST CANCER.** J. G. Roberts, M. Wisbey, K. G. Leach and M. Baum, Departments of Surgery and Medical Physics, Welsh National School of Medicine and the University Hospital of Wales.

Gamma camera images of the spleen obtained following the intravenous injection of heat damaged, autologous, $^{99}Tc^{m}$ labelled red cells provide a means of estimating spleen size in patients.

A new method of calculating spleen size has been validated in 10 patients. The computed weight correlated well with the exsanguinated weight of the excised spleens ($r = 0\cdot9894$) with 95% confidence limits of less than 50 g. This technique has been applied to 40 patients with breast cancer and 5 controls. The cancer patients had significantly larger spleens ($P < 0\cdot05$) and spleen size varied with clinicopathological stage. In 10 patients spleen size has been estimated at primary treatment and some 6 months later. All patients except one showed a reduction in spleen size ($P < 0\cdot01$).

These results suggest that in man, as in experimental animals, splenomegaly occurs as a component of the host response to cancer.